# Impact of obesity on airway remodeling in asthma: pathophysiological insights and clinical implications

**DOI:** 10.3389/falgy.2024.1365801

**Published:** 2024-03-18

**Authors:** Aditya Sri Listyoko, Ryota Okazaki, Tomoya Harada, Genki Inui, Akira Yamasaki

**Affiliations:** ^1^ Division of Respiratory Medicine and Rheumatology, Department of Multidisciplinary Internal Medicine, Faculty of Medicine, Tottori University, Yonago, Japan; ^2^ Pulmonology and Respiratory Medicine Department, Faculty of Medicine, Brawijaya University-Dr. Saiful Anwar General Hospital, Malang, Indonesia

**Keywords:** adipocytokines, airway remodeling, asthma, obesity, outcome

## Abstract

The prevalence of obesity among asthma patients has surged in recent years, posing a significant risk factor for uncontrolled asthma. Beyond its impact on asthma severity and patients' quality of life, obesity is associated with reduced lung function, increased asthma exacerbations, hospitalizations, heightened airway hyperresponsiveness, and elevated asthma-related mortality. Obesity may lead to metabolic dysfunction and immune dysregulation, fostering chronic inflammation characterized by increased pro-inflammatory mediators and adipocytokines, elevated reactive oxygen species, and reduced antioxidant activity. This chronic inflammation holds the potential to induce airway remodeling in individuals with asthma and obesity. Airway remodeling encompasses structural and pathological changes, involving alterations in the airway's epithelial and subepithelial layers, hyperplasia and hypertrophy of airway smooth muscle, and changes in airway vascularity. In individuals with asthma and obesity, airway remodeling may underlie heightened airway hyperresponsiveness and increased asthma severity, ultimately contributing to the development of persistent airflow limitation, declining lung function, and a potential increase in asthma-related mortality. Despite efforts to address the impact of obesity on asthma outcomes, the intricate mechanisms linking obesity to asthma pathophysiology, particularly concerning airway remodeling, remain incompletely understood. This comprehensive review discusses current research investigating the influence of obesity on airway remodeling, to enhance our understanding of obesity's role in the context of asthma airway remodeling.

## 1 Introduction

Overweight and obesity continue to pose substantial global health challenges, impacting individuals across all age groups, irrespective of gender. Alarmingly, the prevalence of adult obesity and severe obesity is poised to persist in its upward trajectory, instigating significant public health concerns due to their association with various chronic health conditions. Analysis of the Global Burden of Disease data has unveiled a disturbing trend, wherein the number of global deaths and disability-adjusted life years (DALYs) attributed to elevated body mass index (BMI) has exhibited a substantial increase from 1990 to 2017. The number of worldwide deaths associated with high BMI rose from around 1.2 million in 1990 to 2.4 million in 2017 for females, and from approximately 1.0 million in 1990 to 2.3 million in 2017 for males. Additionally, the global DALYs associated with high BMI increased from about 33.1 million in 1990 to roughly 70.7 million in 2017 for females, and from about 31.9 million in 1990 to approximately 77.0 million for males (1). Notably, predictive model studies applied in several countries have forecasted a significant increase in obesity, projecting that nearly one in two adults in the US population will be affected by 2030 (2). The projected prevalence is expected to rise from 20.3% to 29.6% in the Brazilian population (3). Moreover, the prevalence is anticipated to more than double in the adult Indian population between 2010 and 2040 (4). These statistics underscore the urgent need for comprehensive global efforts to combat and mitigate the escalating health and societal consequences associated with overweight and obesity.

Addressing asthma-related phenotypes is essential for effective asthma management. Recent evidence has revealed the existence of distinct phenotypes across all age groups (5). Assessing the asthma phenotype correctly presents potential advantages in tailoring personalized treatments, particularly in the administration of biologic agents (6). Obesity has emerged as one of the asthma phenotypes (7), and its association with asthma represents a compelling area of research and clinical interest. Numerous studies have consistently demonstrated a strong association between obesity and asthma, as individuals with overweight and obese exhibit an elevated prevalence and risk of developing asthma (8–12). Remarkably, there is a concerning rise in the prevalence of obesity among individuals with asthma. For example, data from participants with asthma in the US population indicates a shift from 34.3% being overweight and 24.7% being obese in 1999 to 28.8% being overweight and 41.1% being obese in 2016 (13). A multicenter retrospective study in Saudi Arabia in 2022 revealed that among individuals with asthma, the prevalence of overweight and obesity was 31.1% and 46.0%, respectively (14). Beyond merely serving as a risk factor for increased asthma prevalence, obesity exerts a profound influence on asthma's clinical outcomes. Moreover, obesity is increasingly recognized as a potential contributor to the development of airway remodeling in individuals with asthma.

Airway remodeling, a critical phenomenon in the context of respiratory diseases, particularly asthma, involves structural alterations in the airway that have profound implications for both pathological changes and disruptions in normal physiological functions. These structural changes encompass various facets, including epithelial damage, goblet and mucus cell hyperplasia leading to mucus overproduction, subepithelial fibrosis contributing to heightened airway stiffness, hypertrophy of airway smooth muscle (ASM) resulting in increased airway constriction, and structural alterations in the airway vasculature (15–17). Given the multifaceted nature of airway remodeling and its detrimental consequences, understanding the intricate interplay between obesity and airway remodeling in asthma is of paramount importance. It is conceivable that obesity may impact not only the epithelial structure but also contribute to systemic inflammation and the inflammatory milieu of the airways, subepithelial architecture, airway smooth muscle (ASM) function, and the components of the airway vasculature. This review aims to offer insights derived from both laboratory and clinical investigations, seeking to unravel the complex mechanisms connecting obesity and airway remodeling in asthma. These endeavors are essential for advancing our understanding of this intricate relationship, with the potential to inform strategies for enhanced clinical management and therapeutic interventions.

## 2 The impact of obesity on asthma outcome and airway remodeling

Numerous studies have been conducted to comprehend the impact of obesity on the clinical outcomes of individuals with asthma, consistently revealing that obesity plays a pivotal role in exacerbating asthma severity and contributes to a spectrum of adverse clinical outcomes (Table 1). Obesity is associated with increased asthma severity (11) and heightened respiratory symptoms (18), thereby elevating the risk of exacerbations and hospitalization in adults with asthma (19, 26, 27). Additionally, obesity has been significantly linked to increased all-cause and cardiovascular mortality among adults with asthma (20).

**Table 1:** The impact of obesity on clinical outcomes in asthma.

Author, year	Research type	Sample size and group		Outcomes
		Study group	Control group	
Barros et al., 2017 (11)	Population-based study	32.644 adult participants	n/a	• ↑ asthma incidence: OR 4.46 (95% CI: 4.30–4.62) • ↑ current severe asthma for obese class I, II, and III: OR 1.36 (95% CI: 1.32–1.40), 1.50 (95% CI: 1.45–1.55), and 3.70 (95% CI: 3.46–3.95), respectively
Klepaker et al., 2021 (18)	Cross-sectional study	168 overweight/obese asthma participants	228 normal-weight asthma participants	• ↑ symptoms score: OR 1.70 (95% CI: 1.20–2.40) • ↓ pre-BD FEV1 and FVC: OR −2.9 (95% CI: −5.1 to −0.7); −5.2 (95% CI: −7.0 to −3.4), respectively • ↓post-BD FEV1 and FVC: OR −2.8 (95% CI: −4.9 to −0.7); −4.2 (95% CI: −6.1 to −2.3), respectively
Hasegawa et al., 2014 (19)	Multicenter chart review study	607 obese asthma patients	281 normal-weight asthma patients	• ↑ risk of hospitalization of adult asthma patients presenting to ED: OR 1.69 (95% CI: 1.02–2.81); in severely obese patients: OR 1.95 (95% CI: 1.13–3.34)
Sturesson et al., 2023 (20)	Population-based cohort study	328 obese asthma patients	940 normal-weight asthma patients	• ↑ all-cause and cardiovascular mortality: HR 1.26 (95% CI: 1.03–1.54); 1.43 (95% CI: 1.03–1.97), respectively
Huang et al., 2021 (21)	Retrospective longitudinal study	949 obese asthma patients	3417 normal-weight asthma patients	• Worsening pulmonary function test parameters: ↓ FVC (adjusted coefficients (*β*) −0.11 L (95% CI: −0.14, −0.08); ↓ FVC% (*β*: −1.91% (95% CI: −2.64, −1.19) • Annual change of BMI (ΔBMI/year) is significantly associated with accelerated FVC decline (β of ΔFVC/year and ΔFVC %/year for asthmatics: −0.038 L (−0.054, −0.022) and −0.873% (−1.312, −0.435) respectively
Thompson et al., 2021 (22)	Meta-analysis	20.350 obese asthma	11.294 normal-weight asthma	• ↑ asthma medications, SABA OR 1.75 (95% CI: 1.17–2.60); maintenance OCS OR 1.86, (95% CI: 1.49–2.31); higher ICS mean difference 208.14 (95% CI: 107.01–309.27)
Sharma et al., 2008 (23)	Observational cohort study	337 obese asthma patients	524 normal-weight asthma patients	• ↑ AHR risk OR 1.37 (95% CI 1.03–1.82) and the OR is increased with the cut of BMI ≥ 35 and ≥ 40 kg/m^2^ • AHR risk is increased by 3.1% of one-unit increased BMI (1 kg/m^2^).
Sposato et al., 2018 (24)	Retrospective study	77 obese asthma patients were being treated with omalizumab	110 normal-weight asthma patients were being treated with omalizumab	• Obesity class I was identified as a significant risk factor for exacerbations OR 3.11 (95% CI: 1.51–6.42), partial/uncontrolled symptoms OR 2.67 (95% CI: 1.06–6.68), overuse SABA OR 4.45 (95% CI: 1.84–10.77), reduced increase in FEV1 (*β* = −6.98), FVC (*β* = −11.69) and ACT (*β* = −1.84) after omalizumab therapy • Obesity class II was identified as a significant risk factor for exacerbations and for an unchanged/increased level of therapy OR 3.19 (95% CI 1.00–10.33), a reduced response of ACT (*β* = −2.585).
Yang et al., 2018 (25)	Prospective study	50 asthma patients	n/a	• Significant correlations were found between visceral fat area and both airway lumen diameter and lumen area (*r* = −0.35 and *r* = −0.34, respectively); subcutaneous fat area and both wall area and total area (*r* = 0.38 and *r* = 0.34, respectively).

pre-BD, pre-bronchodilator; FEV1, forced expiratory volume in 1 s; FVC, forced vital capacity; ED, emergency department; BMI, body mass index; AHR, airway hyperresponsiveness; FeNO, fractional exhaled nitric oxide; ICS, inhaled corticosteroid; OCS, oral corticosteroid; SABA, short-acting β2 agonists; ACT, asthma control test.

Obesity has consistently been shown to have an adverse impact and is associated with an accelerated decline in lung function, as evidenced by numerous studies (14, 18, 21, 28). Patients with asthma and obesity are more likely to use asthma medications, including short-acting β2-agonists, higher doses of inhaled corticosteroids (ICS), and maintenance oral corticosteroids, compared to normal-weight subjects (22). Additionally, they are less likely to achieve well-controlled asthma despite using ICS or ICS plus long-acting β-agonists (29, 30) and may diminish the effectiveness of biological agents for severe asthma (24). The obese condition is also associated with airway hyperresponsiveness (AHR) (23, 31). However, the impact of obesity on AHR in patients with asthma and obesity remains a topic of ongoing debate, as evidenced by several studies that show no significant difference in AHR or note a negative correlation between obesity and the intensity of AHR (32–34).

The impact of obesity on asthma outcomes in clinical settings is well-established. Guidelines recognize obesity as a risk factor for the development of uncontrolled asthma and an increased risk of future exacerbations (7). This awareness is crucial for clinicians when managing patients with asthma and obesity as comorbidity. The recognition of this connection emphasizes the need for comprehensive care that not only addresses the typical aspects of asthma but also considers the unique challenges and factors associated with obesity. Nevertheless, the influence of obesity on airway remodeling, especially concerning asthma, remains incompletely understood. To date, there exists a critical gap in scientific understanding, as no conclusive examination has been established with empirical evidence to predict or evaluate the progression of airway remodeling in the clinical setting. Several limitations are associated with conducting clinical studies in this regard, which need to be carefully considered. The gold standard for assessing histopathological alterations related to airway remodeling involves direct evaluations of airway structures and lung tissues obtained through surgical samples, bronchial biopsies, or post-mortem analyses (16, 35–37). Post-mortem assessment revealed that the adipose tissue area within the airway wall was positively correlated with BMI. Additionally, it was related to both airway wall thickness and the number of inflammatory cells, providing evidence that obesity may contribute to airway remodeling in asthma (35).

Invasive methods offer valuable insights into the structural alterations occurring in the airways but are often accompanied by ethical and practical challenges. However, non-invasive approaches, including a computed tomography (CT) scan and a biomarker-based approach, may be used to estimate the occurrence of airway remodeling in obesity. A computed tomography examination assesses airway parameters such as bronchial wall thickness (WT), luminal diameter (LD), lumen area (LA), wall area (WA), total area (TA), and the percentage of wall area to total area (WA/TA or wall area %). These parameters can be correlated with fat area measurements, including visceral (VFA), subcutaneous (SFA), and total (TFA) fat areas (25, 38–41), making it a valuable tool for assessing airway remodeling.

The CT approach may currently be the optimal choice for assessing airway remodeling in patients with asthma and obesity. The CT examination revealed that WA% correlated with a higher BMI and a greater volume of adipose tissue, particularly in subcutaneous mediastinal adipose tissue (42). In the asthma population, significant correlations were observed between VFA and both LD and LA, as well as correlations between SFA and both WA and TA (25). These findings suggest that adipose tissue parameters are associated with both bronchial luminal narrowing and bronchial wall thickening.

Biomarker-based approaches may serve as an alternative option to assess and monitor the incidence of airway remodeling in patients with asthma and obesity. To date, there has been no comprehensive study on a biomarker for evaluating airway remodeling in asthma and obesity. Distinct plasma biomarkers were identified in asthma subjects with lean body mass compared to individuals with overweight/obesity, including β-nerve growth factor (β-NGF), interleukin 10 (IL-10), and matrix metalloproteinase 10 (MMP10), which were exclusively associated with asthma subjects with lean body mass. On the other hand, C-C motif chemokine ligand 20 (CCL20), fibroblast growth factor 19 (FGF19), IL-5, leukemia inhibitory factor (LIF), tumor necrosis factor ligand superfamily member 9 (TNFRSF9), and urokinase-type plasminogen activator (uPA) were associated only with individuals with overweight/obesity (43), but these were not found to be associated with airway remodeling. Several studies have proposed such as galectin-3 (44, 45), chitinase-like protein YKL-40 (46, 47), periostin (48), and sestrin-2 (49, 50) as potential predictors of airway remodeling. Nevertheless, research has exclusively focused on individuals with asthma, and currently, no investigations have been undertaken on individuals with both asthma and obesity. This gap in research should be filled by conducting studies that utilize these biomarkers in individuals with asthma and obesity, establishing correlations with the evaluation of airway remodeling through CT scans or bronchial biopsy assessments.

Taken together, obesity has been firmly established as a risk factor for unfavorable clinical outcomes in asthma. While invasive methods are not currently considered clinically practical for the assessment of airway remodeling in asthma and obesity, non-invasive approaches such as the evaluation of CT scans and biomarkers are emerging as potential tools for predicting airway remodeling in these conditions. The utilization of these non-invasive methods holds promise for providing valuable insights into the structural changes occurring in the airways. Nevertheless, the clinical application of these modalities requires additional thorough investigation to establish their efficacy, reliability, and relevance in routine clinical practice.

## 3 The relationship between obesity and genetic predisposition, as well as genes related to airway remodeling

The potential association between obesity and airway remodeling, particularly concerning genetic predisposition, is a subject of consideration. The ongoing investigation into the genetic factors influencing airway remodeling in asthma-obesity is currently limited. Nevertheless, it is crucial to underscore the importance of evaluating the role of genetic predisposition in the context of obesity-induced airway remodeling in asthma. Several genetic predispositions are associated with airway remodeling, including urokinase plasminogen activator receptor (*PLAUR*) (51), *IL-13* (52, 53), and chitinase 3-like 1 (*CHI3L1*) (54). These genetic predispositions may play significant roles in influencing structural changes in the airways. The expression of PLAUR significantly increased in the bronchial epithelium (55). Moreover, the evidence, obtained through the analysis of genetic polymorphisms in *PLAUR* from bronchial biopsy samples of patients with asthma, suggests a potential role in contributing to airway remodeling in asthma by influencing epithelial function (51). Concerning *IL-13* genetic predisposition, an endobronchial biopsy sample indicated an association between genetic variations in *IL-13* and the thickness of the subepithelial layer (53) and genetic variations in *CHI3L1* were correlated with severe asthma and increased YKL-40 expression in the airways, implying a potential link to airway remodeling (54). Among the three genetic predispositions associated with airway remodeling, *PLAUR,* and *CHI3L1* are potentially linked to obesity.

Individuals with obesity exhibited notably higher PLAUR expression in white adipose tissue, upregulated both mRNA expression and plasma levels of soluble PLAUR, positively correlating with metabolic parameters, including BMI (56, 57). Furthermore, individuals with obesity showed significantly higher plasma CHI3L1 concentrations compared to their non-obese counterparts (58). The single nucleotide polymorphism (SNPs) rs883125 in the *CHI3L1* gene is connected to abdominal obesity. Additionally, the research examined the heightened expression of the *CHI3L1* gene in adipose tissue of obese mice. It was observed that pro-inflammatory cytokines enhance *CHI3L1* expression in adipocytes, suggesting a strong association between *CHI3L1* and obesity, as well as obesity-induced pro-inflammatory responses (59). Chitinase 3-like 1 is expressed in both white adipose and lung tissues, and its expression is notably increased by high-fat diet (HFD) in both of these tissue compartments. It plays a crucial role in the accumulation of visceral fat and is associated with the production of cytokines by adipocytes in animal models (60). Furthermore, in humans, both serum and sputum CHI3L1 levels are positively and independently correlated with truncal adiposity (60). These studies suggest a potential association between obesity and genetic predisposition-related airway remodeling.

The observation of a bioactive lipid, sphingosine-1-phosphate (S1P), demonstrated potent regulation of the expression of several genes involved in the control of cell proliferation and airway remodeling (*HBEGF, TGFB3, TXNIP, PLAUR, SERPINE1, RGS4*) (61). Remarkably, circulating S1P levels were found to be elevated in both obese mouse models and individuals with obesity compared to lean and healthy controls, correlating with metabolic abnormalities such as adiposity and insulin resistance (62). This correlation highlights the significance of elevated S1P in individuals with obesity potentially regulating genes associated with airway remodeling. This study potentially provides insights into the assessment of genetic predisposition in obesity-related airway remodeling. Exploring proteins that regulate gene expression and targeting specific genes and proteins could potentially serve as a preventive measure against the development of airway remodeling.

The association between genetic predisposition and obesity-related airway remodeling intricately involves the role of adipokines. Specifically, leptin has been identified as a factor that induces *MUC5AC* expression by activating extracellular signal-regulated kinase 1/2 (ERK1/2) and p38 mitogen-activated protein kinases (MAPKs) (63). In another study, resistin was found to induce *MUC5AC* and *MUC5B* expression through the ERK1/2, p38, and nuclear factor kappa B (NF-κB) signaling pathways (64). These observations carry significant implications, as *MUC5AC* and *MUC5B* are associated with goblet cell metaplasia (65, 66), highlighting the role of adipokines in upregulating specific genes associated with airway remodeling in individuals with obesity.

In summary, obesity is linked to a genetic predisposition and genes associated with airway remodeling. This connection involves the participation of adipokines and proteins that regulate gene-associated airway remodeling. However, research on this aspect is currently limited, and airway remodeling, being a complex alteration, extends beyond changes in goblet cells or epithelial structures. Therefore, conducting research that observes the factors promoting genetic predisposition between obesity and asthma-related airway remodeling becomes crucial.

## 4 The association between asthma, obesity, and alterations in airway structure

**Figure 1:**
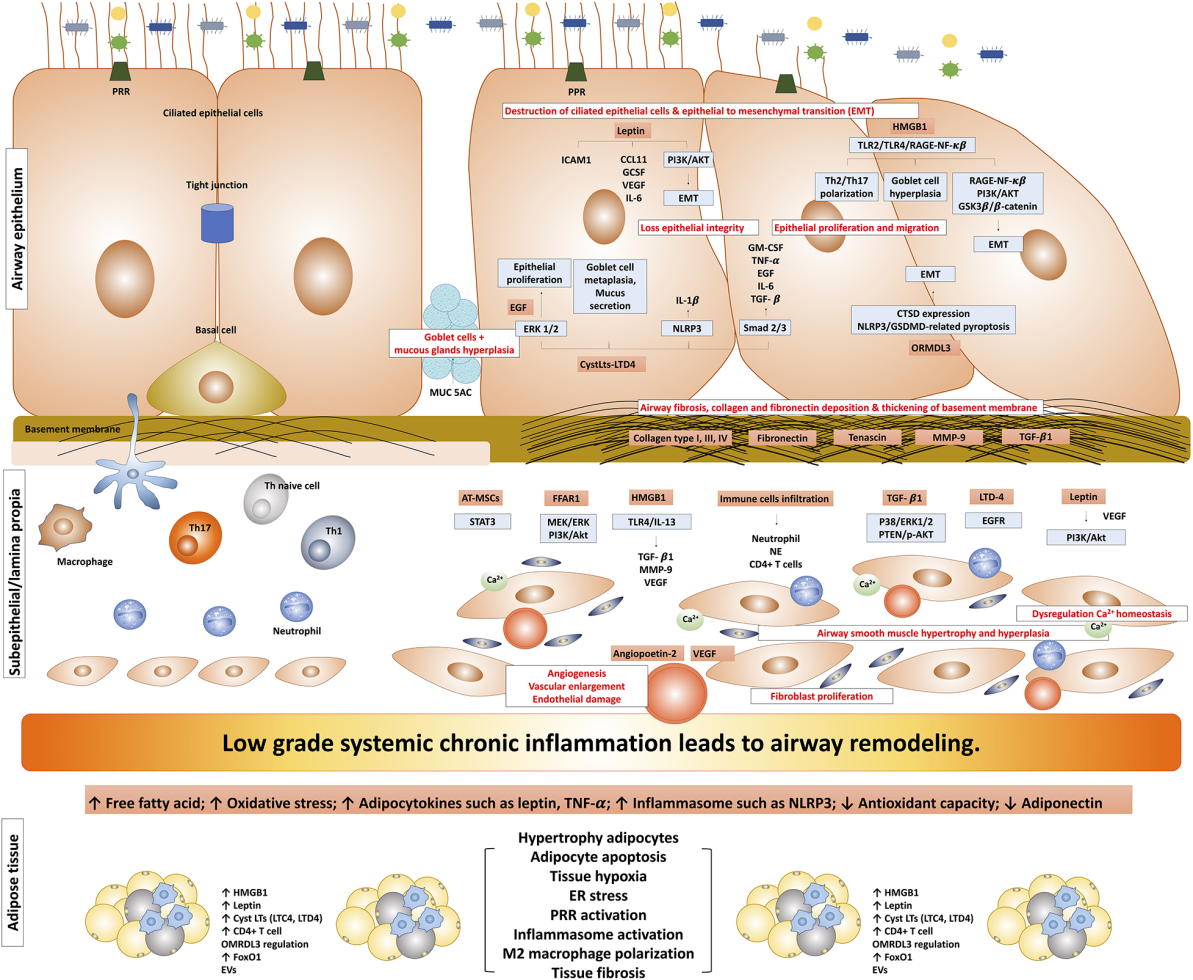
The possible mechanism of obesity-induced airway remodeling in obese-asthma. TNF-α, tumor necrosis factor-α; EGF, epidermal growth factors; GCSF, granulocyte colony-stimulating factor; NLRP3, NLR family pyrin domain containing 3; CTSD, cathepsin D; GSDMD, gasdermin-D; NE, neutrophil elastase; CysLts, cysteinyl leukotrienes; LTD4, leukotriene D4; LTC4, leukotriene C4; ORMDL3, ORMDL sphingolipid biosynthesis regulator 3; HMGB1, high mobility group box 1; TGF-β1, transforming growth factor-β1; ILC, innate lymphoid cells; PRR, pattern recognition receptors; MMP9, matrix metallopeptidase 9; EMT, epithelial-mesenchymal transition; VEGF, vascular endothelial growth factor; EGFR, epidermal growth factor receptor; ASM, airway smooth muscle; ERK, extracellular signal-regulated kinase; AT-MSCs, adipose tissue-derived mesenchymal stem/stromal cells; STAT3, signal transducer and activator of transcription 3; FFAR1, free fatty acid receptor 1; RAGE-NF-κB, receptor for advanced glycation end-products; PI3K/Akt/GSK3β/β-catenin, phosphatidylinositol 3′ -kinase/Akt/glycogen synthase kinase 3β/β-catenin; FoxO1, forkhead box protein class O type 1; EVs, extracellular vesicles.

Airway remodeling induced by obesity represents a complex interplay of various mechanisms involving adipose tissue and inflammation, which significantly impacts asthma pathophysiology. This phenomenon is characterized by structural alterations in the airway, involving changes in various components such as the airway epithelium, sub-epithelium, airway smooth muscle, and vasculature (Figure 1). The accumulation and alterations in adipose tissue may result in a state of chronic, low-grade systemic inflammation. In the context of asthma, the airway inflammatory milieu adds another layer of complexity; these combined effects further enhance the alterations in airway structure, potentially leading to airway remodeling.

### 4.1 Obesity-associated airway epithelial remodeling

Airway epithelial cells play a crucial role in maintaining the integrity of the air passages and safeguarding the lungs from inhaled environmental threats (67, 68). Airways are composed of various cell types, including ciliated epithelial cells, mucous or secretory cells (such as goblet cells and mucus-producing cells), basal cells, and club cells, previously known as clara cells (69–71). Ciliated airway epithelial cells are pivotal for preserving the integrity of the mucosal barrier through mucociliary clearance (70, 72). The mucus serves as a crucial component in the lungs’ defense against airborne particles (73, 74). Club cells play a primary role as secretory cells, maintaining the basal cell-deficient epithelium in the distal bronchial and bronchiolar airways (75, 76). Airway basal stem cells play a crucial role in tissue regeneration and innate immunity (77–79). The diverse roles played by these various cell types underscore the intricate and vital functions of the airway epithelium in preserving respiratory health and protecting against environmental threats.

The pathological observation of airway remodeling within the epithelial airway is a significant indication of structural changes including loss of epithelial integrity leading to epithelial-mesenchymal transition (EMT), destruction of ciliated cells, a decrease in EMT-related epithelial markers, and an increase in EMT-related mesenchymal markers, goblet cell and mucous gland hyperplasia and metaplasia, an increase in the production of extracellular matrix (ECM) components like fibronectin, collagen types III and V, and MMP-9 within the reticular basement membrane, and upregulation of growth factor release and the overexpression of receptors, such as epidermal growth factor receptors (EGFR) (80–83).

One promising avenue for exploring the impact of obesity on epithelial airway remodeling is to investigate its potential association with high mobility group box protein 1 (HMGB1). It is a nuclear protein with the unique ability to act as an alarmin, triggering the activation of the innate immune system and orchestrating various physiological and pathological responses, particularly chronic inflammation (84). Notably, individuals with obesity often exhibit elevated expression of HMGB1 in adipose tissue, as well as elevated levels of plasma HMGB1 (85–87). In individuals with asthma, levels of HMGB1 in induced sputum were significantly elevated, and plasma HMGB1 levels were notably higher in patients with moderate and severe asthma when compared to their healthy counterparts (88, 89), suggesting that HMGB1 may play a role in initiating and perpetuating chronic inflammatory processes in both obesity and asthma. Beyond its role in airway inflammation, HMGB1 is also associated with mucus production and regulates airway inflammation by directly influencing naive T cells, inducing Th2 and Th17 polarization through toll-like receptors-2 (TLR2), TLR4, and the receptor for advanced glycation end-products (RAGE)-NF-κB signaling pathways in the asthma mice model (90). The administration of HMGB1 resulted in pronounced goblet cell hyperplasia and excessive mucus secretion, which could be mitigated with anti-HMGB1 treatment (91). Furthermore, HMGB1 has been demonstrated to play a role in promoting EMT. High mobility group box protein 1 contributes to EMT by reducing the expression of epithelial markers such as ZO-1, occludin, VDR, and E-cadherin while concurrently increasing the expression of mesenchymal markers, including vimentin, N-cadherin, CTGF, SNAI2, and fibronectin (92, 93). The precise mechanism through which HMGB1 induces EMT in asthma remains unclear, but it may potentially involve RAGE and the phosphatidylinositol 3′ -kinase/Akt/glycogen synthase kinase 3β/β-catenin (PI3K/Akt/GSK3β/β-catenin) signaling pathway (92) or the RAGE/NF-κB signaling pathway (93). These findings suggest that HMGB1 could play a significant role in the intricate interaction between obesity and asthma-related airway remodeling, especially concerning airway inflammation, mucus production, and EMT.

Leptin, a hormone primarily secreted by adipose tissue, presents another intriguing avenue of investigation when exploring the relationship between obesity and airway epithelial remodeling. Leptin levels are significantly higher in individuals with obesity compared to those with normal weight, and a substantial correlation exists between leptin levels and BMI (94, 95). Leptin levels are markedly increased in individuals with asthma compared to those who have never had asthma (96) and several studies have reported higher plasma leptin in patients with asthma and obesity in comparison to their normal-weight counterparts, with even greater elevation seen in individuals with severe asthma (97–99). Lung tissue and bronchial epithelial cells express the leptin receptor, which plays a substantial role in stimulating epithelial cell proliferation (100, 101). Leptin has been found to exert various effects on epithelial cell lines, including the induction of intercellular adhesion molecule 1 (ICAM-1) expression, increased production of inflammatory mediators such as CCL11, granulocyte colony-stimulating factor (G-CSF), vascular endothelial growth factor (VEGF), and IL-6, promotion of cell migration, inhibition of apoptosis, and stimulation of epithelial cell proliferation (102). Leptin may also contribute to alterations in EMT. Leptin has been found to elevate the levels of the mesenchymal phenotype marker including vimentin and fibronectin (103–105), downregulate epithelial phenotype marker E-cadherin (104, 105), and enhance the expression of EMT-related transcription factors, including β-catenin, Twist, ZEB-1 (103, 106). The precise mechanism by which leptin induces EMT in asthma remains unclear, but it may involve the activation of the ERK signaling pathway (103) or PI3K/Akt-dependent pathway (105). According to these findings, leptin has a potential role in obesity-induced epithelial remodeling by triggering epithelial proliferation and promoting EMT.

Emerging evidence suggests that cysteinyl-leukotrienes (CysLTs), a group of lipid mediators produced during the arachidonic acid metabolism via the 5-lipoxygenase pathway (107), may contribute significantly to the pathogenesis of obesity-induced epithelial airway remodeling. Within this class of bioactive molecules, two distinct categories can be discerned: leukotriene B4 (LTB4) and CysLTs, consisting of LTC4, LTD4, and LTE4. Leukotriene B4 is a potent chemoattractant, and stands as a formidable mediator of inflammation, orchestrating the recruitment of immune cells to sites of injury or infection. On the other hand, the CysLTs—LTC4, LTD4, and LTE4—exert their influence as formidable bronchoconstrictors, assuming a paramount role in the pathogenesis of asthma (108). The differentiation in their functions underscores the complexity of leukotrienes and their diverse impact on various physiological and pathological processes in asthma. Clinical investigations have provided compelling evidence regarding the pivotal role of CysLTs in the intricate process of airway remodeling, as observed by the significant correlation between CysLTs in exhaled breath condensate and the thickness of the reticular basement membrane, as measured in endobronchial biopsies (109). Additionally, the therapeutic potential of targeting CysLTs to alleviate airway remodeling has been demonstrated. Specifically, the administration of the cysteinyl leukotriene1 (CysLT1) receptor antagonist, montelukast, effectively reduced mucus accumulation and subepithelial fibrosis in a murine model sensitized and challenged with ovalbumin (OVA) (110). Leukotriene D4 induced the driving of epithelial cell proliferation via increased phosphorylation of ERK1/2, influenced the expression of CysLT receptors in airway epithelial cells, and resulted in the phosphorylation of EGFR. These signaling cascades culminated in the upregulation of c-myc expression. Notably, the presence of growth factors such as epidermal growth factor amplified the effect of LTD4, highlighting the potential for synergistic interactions in promoting airway epithelial cell proliferation (111). Furthermore, it has been observed that LTD4 contributes to the induction of airway inflammation by activating the NOD-like receptor thermal protein domain associated protein 3 (NLRP3) inflammasome (112). The influence of leukotrienes on EMT in the context of epithelial airway remodeling remains uncertain. However, one study observed that the stimulation of LTD4 increased remodeling markers, such as vimentin and MUC5AC, through the transforming growth factor-β (TGF-β)/smad2/3-mediated pathway (112). Based on evidence that leukotriene production increases in individuals with asthma and obesity compared to individuals with normal or pre-obese asthma (113), and individuals with asthma and obesity exhibit elevated levels of CysLTs in exhaled breath condensate compared to their non-obese counterparts (114), then leukotrienes may have a potential role in obesity-induced epithelial airway remodeling. Leukotrienes also play a role in adipogenesis and the inflammatory processes within adipocyte tissue. During adipogenesis, the production of LTC4 and other CysLTs increases. LTC4, in particular, has been found to enhance adipogenesis by activating CysLT1 receptors in 3T3-L1 cells, leading to elevated intracellular triglyceride levels (115). Adipocytes secrete leukotrienes that may be related to obesity-associated inflammation. Subcutaneous and epididymal adipose tissue increase the production of LTB-4 and CysLTs in the HFD mice model compared to the control. The measurement of leukotriene secretion by 3T3-L1 adipocytes revealed that adipocytes secrete large amounts of leukotrienes. Furthermore, leukotrienes play a crucial role in macrophage and T-cell chemotaxis induced by 3T3-L1 (116). According to these findings, obese-induced epithelial remodeling may be related to the increased production of CysLTs in the airway and the elevated secretion of leukotrienes by adipose tissue. This leukotriene production is suggested to impact the alteration of the epithelial airway.

Another intriguing factor that has emerged in the context of obesity-induced epithelial airway remodeling is ORM1-Like Protein 3 (ORMDL3) and 17q21-related genes. ORM1-Like Protein 3 belongs to the Orm protein family and encodes a transmembrane protein with a critical role in regulating sphingolipid synthesis (117). The gene cluster comprising ORMDL3 and gasdermin B (GSDMB) is located on chromosome 17q21 and is strongly associated with asthma (118). This chromosome may play a crucial role in the pathological connection between asthma and obesity. Among asthmatics, seven SNPs clustered in 17q21.2 were identified as being associated with higher BMI (119). Further investigation is needed to understand the genetic susceptibility mechanism of 17q21, which may lead to an increased expression of mucosal GSDMB; elevated mucosal GSDMB expression is linked to a cell-lytic immune response, ultimately compromising airway immunocompetence (120). A case-control association study conducted in the Japanese population observed that *ORMDL3/GSDMB* is an important susceptibility gene for early-onset adult asthma (121). A notable increase in ORMDL3 expression, increased expression of epithelial-mesenchymal transition (EMT)-related markers, including E-cadherin, vimentin, and fibroblast-specific protein 1 (FSP-1), and a positive correlation with the severity of airway remodeling was observed in the OVA-induced mice model (122). This suggests that ORMDL3 may indeed play a critical role in the regulation of EMT within the bronchial epithelium. Elevated protein levels of ORMDL3 were also observed in mice models exhibiting both asthma and obesity. Additionally, this study observed heightened expression of NLRP3, GSDMD, and cathepsin D (CTSD) in human bronchial epithelial cells infected with lentivirus overexpressing ORMDL3. These results suggest that ORMDL3 might modulate pyroptosis and subsequent airway remodeling in asthma associated with obesity through the CTSD/NLRP3/GSDMD pathway (123).

The ECM protein degradation products, sometimes referred to as “matrikines”, may play a role in regulating the epithelial remodeling process, and there is a possibility that matrikines are involved in obesity-induced epithelial remodeling. Obesity induces excessive lipid accumulation in adipocytes, leading to fibrosis, a significant contributor to obesity-related metabolic dysregulation. This fibrosis results in excess deposition of ECM components such as collagens, elastin, and fibronectin (124–126). Subcutaneous adipose tissue exhibits a more pronounced and strong association with the ECM (127, 128), encompassing collagens, proteases, and cell adhesion-related genes. In contrast, visceral adipose tissue is associated with cellular responses to extracellular signals (128). The expression of MMP-9 in subcutaneous adipose tissue, a marker of ECM degradation, is higher in metabolically unhealthy obese individuals compared to metabolically healthy lean individuals (127), suggesting that obesity is more prominently associated with ECM degradation in adipose tissue. One such matrikines is the tripeptide Pro-Gly-Pro (PGP). A study observed that N-acetylated PGP (AcPGP) is elevated in the sputum of patients with severe asthma. Furthermore, PGP neutralization attenuates heightened airway resistance and epithelial remodeling in the house dust mite (HDM) model. This study also observed that the direct application of AcPGP induces epithelial remodeling and mucus production, suggesting that matrikines are involved in the mechanism of epithelial airway remodeling (129). While matrikines may potentially play a role in epithelial remodeling, there is currently no study exploring the direct impact of matrikines derived from adipose tissue, particularly subcutaneous adipose tissue, on airway remodeling. However, considering that obesity leads to the deposition of ECM, the role of matrikines as a potential factor in obesity-induced epithelial airway remodeling should be considered.

The role of IL-4 and interferon- γ (IFN-γ) in obesity-related epithelial remodeling should be considered. Serum levels of IL-4 show a notable increase in individuals with asthma who are overweight or obese compared to those with a normal weight (130). While clinical studies on IFN-γ-related systemic inflammation in subjects with asthma and obesity are limited, *in vivo* studies using obese mice lacking IFN-γ show a decrease in systemic inflammation, suggesting that IFN-γ plays a role in the regulation of systemic inflammation (131). Interleukin-4 and IFN-γ may contribute to the development of epithelial-airway remodeling by inducing transcription factors present in antagonistic and polarized gene regulation networks in epithelial cells (132). However, the exact mechanism by which IL-4 induces epithelial remodeling remains not completely understood. Perhaps, IL-4 induces epithelial remodeling by promoting the secretion of epithelial wingless/integrase 5a (Wnt5a) (133).

Taken together, the complex development of airway epithelial remodeling may be influenced by various factors, including HMGB1, adipocytokines such as leptin, leukotrienes, and ORMDL3. Additionally, the role of matrikines like PGP and cytokines including IL-4 and IFN-γ may also contribute to epithelial alterations in obesity. However, it's essential to recognize that airway epithelial remodeling is not solely driven by these factors but is the result of a multifaceted interplay involving genetics, lung development, asthma severity, and the epithelial response to external factors like allergens, bacteria, or viruses, and factors associated with airway epithelium injury and repair. These intricate mechanisms underscore the need for further research to achieve a comprehensive understanding of this complex process.

### 4.2 The intricate inflammatory processes shared between asthma and obesity may play a key role in the development of airway remodeling

The development of inflammation in asthma and obesity leading to a chronic inflammatory process that results in airway remodeling is a subject of debate. To date, there is no clear evidence supporting chronic inflammation as the exact cause of airway remodeling in these conditions. Clinical studies on airway remodeling in the obese population are still limited. The correlation between airway remodeling and systemic inflammation in asthma is also constrained. Currently, studies only evaluate the development of airway remodeling, such as through CT scans, and correlate it with asthma severity (134) or lung function and risk of exacerbation (135), but the correlation with markers of systemic inflammation remains limited.

Airway inflammation is one of the crucial factors contributing to airway remodeling in asthma. Evidence indicates a correlation between inflammatory markers in the airway and airway remodeling, as assessed by sputum neutrophils, eosinophils, or sputum eosinophil cationic protein, demonstrating a significant correlation with airway thickness on CT examination or basement membrane thickening from biopsy results (38, 136, 137). It's essential to note that the data was collected from individuals with asthma, and there is still limited study available on individuals with both asthma and obesity. While individuals with asthma and obesity seem to exhibit either no significant difference or an inverse relationship with eosinophilic airway inflammation compared to non-obese individuals with asthma (34, 138), evidence of airway inflammation was observed through higher levels of IL-5, IL-17A, and IL-25 mRNA in individuals with both obesity and asthma (139). Nevertheless, individuals with both obesity and asthma exhibit a more pronounced increase in sputum neutrophils compared to those with non-obese asthma (138, 139), although an eosinophilic phenotype also exists in individuals with obesity and asthma (140, 141). This evidence suggests that airway inflammation may be related to airway remodeling development in individuals with obesity and asthma, although no clear study correlates airway inflammation markers with airway remodeling parameters in the population with asthma and obesity.

The development of airway remodeling may not be solely due to local airway inflammation. Systemic inflammation may be associated with the development of airway remodeling, although the data supporting this connection is still limited (142, 143), and there is no clinical study yet in the population with asthma and obesity. Individuals with severe asthma exhibited significantly greater WT% and a higher WA% compared to those with mild-to-moderate asthma and healthy individuals (134). Moreover, they are more likely to display elevated levels of biomarkers associated with systemic inflammation, such as high-sensitivity C-reactive protein (hs-CRP), fibronectin, TNF-α, and IL-8 (144–147). Several systemic inflammatory markers, including serum hs-CRP and periostin, exhibit correlations with airway remodeling parameters assessed by CT (142, 143). These data suggest the role of the systemic inflammatory process in the development of airway remodeling. Given this evidence, this section aims to explore the possibility of inflammation development in obesity-asthma as a risk factor for airway remodeling. Furthermore, it discusses several proteins or biomarkers that may play a role in this process, emphasizing the unmet need that requires investigation in the future.

**Figure 2:**
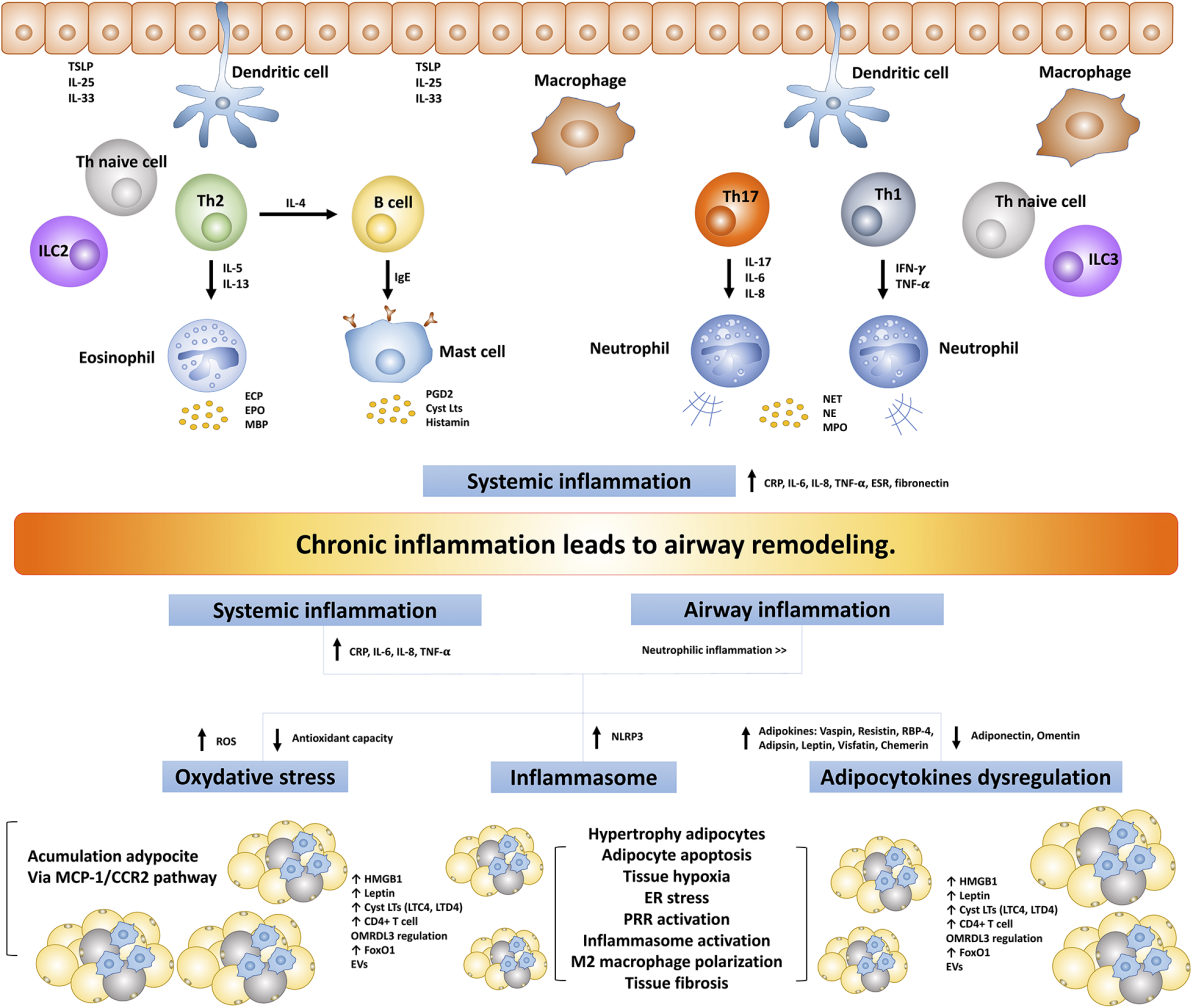
The intricate inflammatory process between asthma and obesity leads to airway remodeling. TSLP, thymic stromal lymphopoietin; ILC, innate lymphoid cells; IFN-γ, interferon-γ; TNF-α, tumor necrosis factor-α; ECP, eosinophil cationic protein; EPO, eosinophil peroxidase; MBP, major based protein; PGD2, prostaglandin D2; CysLts, cysteinyl leukotrienes; NET, neutrophil extracellular traps; NE, neutrophil elastase; MPO, myeloperoxidase; TLR, toll-like receptors; ROS, reactive oxygen species; NLRP3, NLR family pyrin domain-containing protein 3; RBP-4, retinol-binding protein 4; hs-CRP, high-sensitivity C-reactive protein; MCP1/CCR2, monocyte chemoattractant protein 1/CC chemokine receptor 2; ER, endoplasmic reticulum; PRR, pattern recognition receptors; FoxO1, forkhead box protein class O type 1; EVs, extracellular vesicles; ORMDL3, ORMDL sphingolipid biosynthesis regulator 3; HMGB1, high mobility group box 1; ESR, erythrocyte sedimentation rate.

Asthma and obesity, two prevalent and complex health conditions, share an intriguing commonality in the form of chronic inflammation. Despite having distinct clinical presentations, both conditions are characterized by intricate inflammatory processes (Figure 2). Traditionally, asthma is defined by airway inflammation and bronchoconstriction, while obesity is acknowledged as a state of persistent low-grade inflammation driven by the production of pro-inflammatory cytokines and adipokines originating from adipose tissue. This persistent inflammatory milieu doesn't confine itself to adipose tissue but extends its impact on the respiratory system, potentially contributing to airway remodeling. Several mechanisms have been recognized in the development of obesity-induced chronic inflammation including oxidative stress (148, 149), endoplasmic reticulum stress (150, 151), increasing oxygen consumption leading to adipocyte hypoxia (152, 153), inflammasome activation particularly NLRP3 (154, 155), and pattern recognition receptors (PRR) activation and regulation particularly TLR2 and TLR4 activation (156–158).

The complex inflammatory processes common to both asthma and obesity, alongside systemic inflammation, emerge as key contributors to the development of airway remodeling in individuals dealing with the dual challenges of asthma and obesity. Asthma is no longer viewed solely as a localized lung inflammatory condition; instead, it is increasingly recognized as a systemic inflammatory disease. This systemic inflammatory process appears to be closely associated with various aspects of asthma, including its severity, responses to ICS, and the development of comorbidities in individuals with asthma. Numerous studies have consistently demonstrated elevated systemic inflammatory markers in individuals with asthma. These markers include CRP/hs-CRP, fibrinogen, erythrocyte sedimentation rate (ESR), serum amyloid-A, total leukocyte counts, serum TNF-α, and IL-6 (159–163). On the other hand, obesity is not solely characterized by localized inflammation within adipose tissue but also exhibits a systemic inflammatory process. This systemic inflammation has far-reaching implications, as it is closely associated with the development of various complications, and metabolic and systemic diseases associated with obesity (164, 165). Elevations in various inflammatory markers, such as CRP/hs-CRP (166–168), ESR (168), TNF-α (166, 169, 170), IL-6 (169, 171), IL-8 (172, 173), IL-20 (174), have been reported in individuals affected by obesity, which exhibit a notable tendency toward neutrophilic inflammation (138, 175, 176). Furthermore, obesity alone, without asthma or other lung diseases, can exacerbate bronchial inflammation. This is triggered by systemic or local inflammation, as supported by the observation that serum levels of CRP, IL-6, and IL-8 were significantly increased, along with elevated sputum levels of neutrophils, sputum mRNAs of IL-6, IL-8, IL-13, IL-17, and IL-23 (177). Furthermore, this study observed that the sputum expression levels of the prototypic transcription factors of type 1 (T-bet) and type 2 (GATA3) cells were associated with BMI (177). These findings suggest that individuals with obesity experience a more pronounced occurrence of airway and systemic inflammation, potentially contributing to organ damage and the development of airway remodeling.

The first potential key role in the development of chronic inflammation in asthma and obesity, leading to airway remodeling, is the occurrence of oxidative stress. Elevated free fatty acid levels in obesity can trigger endoplasmic reticulum (ER) stress, apoptosis, inflammation, and reactive oxygen species (ROS) production which can progress to oxidative stress (178, 179), potentially playing a significant role in the development of chronic inflammation. Individuals with obesity demonstrate elevated levels of ROS and compromised antioxidant defenses, with the imbalance between oxidants and antioxidants closely correlated with obesity parameters, including BMI (149, 180–183). The oxidative stress related to obesity is generated from NADPH oxidases (Nox) (184, 185) and is associated with an observed increase in oxidative stress parameters, such as malondialdehyde (MDA) and nitric oxide products (NO), coupled with a decrease in the activity of catalase, superoxide dismutase (SOD), and glutathione peroxidase (GPx) (149, 182, 186–190). Elevated hydrogen peroxide levels in the bronchoalveolar lavage fluid and increased serum thiobarbituric acid reactive substances (TBARS) observed in the HFD-OVA mice model suggest that oxidative stress may also play a significant role in asthma with obesity condition (191). Furthermore, in a separate study, it was noted that the mice model asthma and obesity exhibited higher concentrations of MDA compared to the asthma group with a normal BMI, alongside a significant reduction in glutathione (GSH) levels in the obesity-asthma group (192). It is crucial to acknowledge the imbalance between oxidative stress and antioxidant activity in individuals with asthma and obesity, as oxidative stress actively participates in inflammation by both initiating and sustaining the process (193, 194). Furthermore, correlations between markers of oxidative stress and inflammatory markers, such as hs-CRP and IL-6 were observed. Specifically, hs-CRP levels exhibited a positive correlation with free oxygen radicals test (FORT) and MDA, while showing a negative correlation with the activities of catalase, SOD, and GPx. Simultaneously, IL-6 levels demonstrated a negative correlation with the activities of SOD (195, 196). The interaction between oxidative stress and the inflammatory process may play a role in contributing to the development of airway remodeling induced by obesity.

The second potential key role in the development of chronic inflammation that leads to airway remodeling in asthma and obesity is the dysregulation of adipocytokines. Elevating oxidative stress through the NADPH oxidase pathway can trigger the overproduction of adipocytokines, signaling molecules, inflammatory mediators, peptides/proteins, and immune molecules secreted by adipose tissue (197). Individuals with obesity exhibit significantly higher levels of various inflammatory markers and adipocytokines compared to their non-obese counterparts. These elevated adipokine markers include vaspin, resistin, RBP-4, adipsin, leptin, visfatin, chemerin, and several cytokine markers, as previously discussed, including hs-CRP, IL-4, IL-6, IL-8, Monocyte chemoattractant protein-1 (MCP-1), and TNF-α (173, 198–204). Conversely, the levels of adiponectin and omentin are notably lower in individuals with obesity (198, 203–206). Moreover, leptin, a prominent adipocytokine, has been shown to enhance the production of multiple inflammatory factors, including CCL2/MCP-1, eotaxin (CCL11), CXC chemokine ligand 8 (CXCL8)/IL-8, CXCL10/IP-10, and IL-6 (207). Importantly, higher levels of leptin are correlated with increased levels of hs-CRP, IL-6, and TNF-α (198). The role of adipocytokines in enhancing the inflammatory profile in obesity may significantly contribute to the development and progression of systemic inflammation. This, in turn, may lead to the development of airway remodeling in individuals with asthma and obesity.

The third potential key role in the development of chronic inflammation that leads to airway remodeling in asthma and obesity is the role of inflammasome. The NOD-, LRR- and pyrin domain-containing protein (NLRP) inflammasome, a critical component of the innate immune system, is responsible for triggering caspase-1 activation and the release of proinflammatory cytokines (IL-1β/IL-18), following exposure to cellular damage or infection (208). The NLRP inflammasome, with a particular focus on NLRP3, appears to occupy a pivotal role in the intricate interplay of obesity and asthma. This molecular complex plays a central role in regulating inflammatory responses in both of these conditions. Individuals with asthma, particularly those with neutrophilic asthma, exhibited the presence of NLRP3 (209). Airway remodeling may be related to the regulation of NLRP3. A reduction in the production of type 2 inflammation cytokines, along with decreased tissue damage, as evidenced by a reduction in goblet cell hyperplasia, mucus production, and peribronchial and perivascular cell infiltration, was observed in NLRP3-deficient mice (210). In the context of obesity, NLRP3 has been observed to play a significant role in the development of obesity and the associated inflammatory processes. Mice models with deficient inflammasomes, including NLRP3, apoptosis-associated speck-like protein containing a caspase activation and recruitment domain (ASC), and caspase-1, have exhibited protection against HFD-induced obesity. This protection is associated with reduced levels of IL-1β, as well as lower plasma insulin and leptin levels. Moreover, these studies revealed the specific roles of inflammasome components. A caspase activation and recruitment domain appears to contribute to the development of adipocyte hypertrophy, while caspase-1 is essential for the influx of macrophages into adipose tissue under HFD conditions (211). Notably, a clinical study identified heightened expression of NLRP3 and IL-1β, increased IL-1β, elevated adipose tissue macrophages, and a reduced number of regulatory T cells within the visceral adipose tissue of metabolically unhealthy obese individuals in comparison to their healthy obese and lean counterparts (212). These findings collectively suggest that inflammasomes, particularly NLRP3, play a significant role in the pathological processes related to chronic inflammation in obesity, contributing to the development of metabolic and systemic disorders, and may also contribute significantly to the development of obesity-induced airway remodeling.

An additional factor to consider in the context of obesity-induced airway remodeling within asthma-related inflammatory processes is the involvement of anti-inflammatory modulators, such as secretoglobin and IL-37. Secretoglobin family proteins, found in secretory tissues of barrier organs, serve as anti-inflammatory proteins for airway diseases (213). Club cell secretory protein-16 (CC-16), a distinct member of the secretoglobin family, is particularly abundant in normal airway secretions. A significantly higher serum CC-16 level was observed in individuals with asthma compared to their healthy counterparts. Furthermore, decreased CC-16 production was found to contribute to persistent airway inflammation and a decline in lung function (214). In obese conditions, a decrease in the percentage of CC-16-expressing cells in the small airways of mice and humans was observed, and BMI showed a significant correlation with reduced circulating CC-16 levels across all populations, implicating circulatory CC-16 in the association between BMI and clinical asthma (215). The second anti-inflammatory factor is IL-37, which serves as a crucial anti-inflammatory modulator in inflammation induced by obesity. Interleukin 37 mitigated airway remodeling induced by HDM by inhibiting EMT mediated by IL-24 through the ERK1/2 and signal transducer and activator of transcription 3 (STAT3) pathways (216). Interleukin-37 also mitigates diet-induced adipose tissue inflammation by decreasing macrophage and lymphocyte infiltration, along with reducing levels of IL-1β, IL-6, and CXCL1 (217). Considering the pivotal roles of secretoglobin CC-16 and IL-37 as anti-inflammatory modulators, their involvement in the development of obesity-induced airway remodeling in conjunction with the inflammatory process should be acknowledged.

The development of chronic inflammation that leads to airway remodeling in asthma and obesity might not solely arise from the interconnection between these two conditions. Obesity, a known risk factor, exhibits a multifaceted association with various comorbidities, including but not limited to obstructive sleep apnea (OSA) (218), diabetes mellitus (219), and cardiovascular disease (220). Notably, OSA emerges as a pivotal link, intricately connected to both obesity and asthma, thereby amplifying the risk of inflammatory processes. The heightened incidence of OSA in the presence of obesity and asthma is concomitant with an escalation in bronchial and systemic inflammatory markers. Noteworthy among these are IL-6 (221), IL-8 (222–225), neutrophils (222), ICAM-1 (223, 225), vascular cell adhesion protein 1 (VCAM-1) (225), MMP-9 (224), CRP (221, 225, 226), TNF-α (225, 226), E-selectin, and P-selectin (225), as evidenced by multiple studies. Remarkably, there is a noteworthy elevation of leptin in patients with OSA, and this increase demonstrates a correlation with the severity of OSA (227). Leptin, recognized as a pivotal factor in obesity-induced chronic inflammation, assumes a significant role in the intricate interplay of molecular events associated with OSA. The orchestrated elevation of these inflammatory mediators in the milieu of OSA underscores its potential contribution to the development of inflammation, particularly in the context of airway remodeling observed in obese patients with OSA-asthma conditions.

Taken together, the development of chronic inflammatory processes may represent a pivotal component in the airway remodeling observed in individuals affected by both obesity and asthma. Although there is no clear evidence supporting chronic systemic inflammation as the exact cause of airway remodeling in these conditions, there is evidence that systemic inflammation is associated with airway remodeling (142, 143). It is noteworthy that obesity itself can increase bronchial inflammation, triggered by systemic or local inflammation. Factors such as oxidative stress, dysregulation of adipocytokines, the inflammasome, anti-inflammatory modulators, and the presence of comorbidities may be related to the development of inflammation-associated airway remodeling in individuals with asthma and obesity. However, this concept needs further study to achieve a comprehensive understanding of this aspect.

### 4.3 Obesity-induced airway fibrosis

Obesity emerges as a significant risk factor in the induction of fibrosis in lung disease (228, 229). This observation suggests a potential association with the development of fibrosis in the context of obesity-related asthma. Substantial fibrosis, particularly pronounced in the peribronchial and perivascular areas with a lesser extent observed in the lung parenchymal lesion, was noticed in HFD-induced animal models compared to the control group (230). Moreover, collagen deposition in the peribronchial and perivascular regions was also observed in the HFD animal model (231).

Transforming growth factor-β1 is a central cytokine in the process of fibrosis and plays a critical role in mediating the differentiation of fibroblasts into myofibroblasts (232, 233) and increased collagen deposition (233–235). An upregulation in the expression of TGF-β mRNA and protein, specifically TGF-β1, has been observed in both HFD-induced and genetically obese animal models (236–239). Furthermore, the administration of anti-TGF-β antibodies resulted in significant reductions in body weight gain (239), implying a potential association between the pathogenesis of obesity, adipogenesis, and TGF-β signaling. In a clinical study, elevated TGF-β1 levels were observed in patients with overweight and obese compared to those with normal weight (240), while among patients with asthma, an increase in TGF-β1 levels was noted in individuals with moderate to severe asthma compared to those with mild asthma (241). Moreover, in individuals with obesity and insulin resistance, a significant correlation was revealed between the expression of TGF-β1 and the presence of various collagen types, including collagen type I, III, and VI, which was further associated with the extent of fibrosis within white adipose tissue (242). Although the role of TGF-β1 in the context of obesity-induced airway fibrosis in asthma remains elusive, and one study observed no difference in serum levels of TGF-β1 between individuals with asthma and obesity compared to healthy controls (243), the role of TGF-β1 in the context of obesity-induced airway fibrosis should be considered.

Another potential mechanism underlying obesity-induced airway fibrosis in asthma involves MMPs. The increased expression of MMP-9 was found to be associated with collagen III, collagen V, and tenascin deposition in the basement membrane of asthma individuals (244). Elevated concentrations of exhaled MMP-9 were identified in individuals with severe asthma when compared to those with mild to moderate (245). Individuals with obesity have also been observed to have an increase in MMP-9 levels, as evidenced by several studies (246–249). Notably, MMP-9 expression showed a positive correlation with BMI and a negative correlation with insulin sensitivity (247). Within the context of obesity-induced airway remodeling, MMP-9 is suggested to be a key player in the development of airway fibrosis.

Forkhead box protein class O type 1 (FoxO1) may play a role in the development of airway fibrosis. Blocking FoxO1 inhibits profibrotic gene expression by macrophages and reverses peribronchial fibrosis in a murine model of allergic asthma (250). Deletion of FoxO1 and genetic ablation of FoxO1 in macrophages significantly decrease interferon regulatory factors (IRF4) and various M2 macrophage-associated genes, suggesting the role of FoxO1 as a regulator of airway inflammation, especially associated with macrophages (251). Obesity-induced airway fibrosis may involve the role of FoxO1. Forkhead box protein class O type 1 is one of the most significant transcription factors, highly expressed in adipocytes, and plays a role in adipogenesis and insulin resistance (252). In individuals with obesity, both the mRNA levels and protein expression of FoxO1 were significantly increased compared to their non-obese counterparts (253). Considering this crucial point, FoxO1 may play a role in obesity-induced airway remodeling.

Taken together, the process of the airway may initiate with an excess of TGF-β1 and MMP-9, often derived from the inflammatory processes occurring in both obesity and asthma. This imbalance can mediate the differentiation of fibroblasts into myofibroblasts and lead to collagen deposition. Moreover, FoxO1 may play a role in contributing to airway fibrosis in individuals with asthma and obesity. However, the development of airway fibrosis is a complex mechanism, requiring additional research to fully understand the precise underlying mechanisms involved in obesity-asthma airway remodeling.

### 4.4 Obesity-associated airway smooth muscle remodeling in asthma

Pathological changes in ASM remodeling include both hypertrophy (an increase in muscle mass) and hyperplasia (an increase in the number of ASM cells) (17). Asthmatic ASM exhibits uniform staining for both smooth muscle-specific contractile proteins α-smooth muscle actin (α-SMA) and calponin and has a higher proliferation rate compared to normal subject (254). The precise mechanism governing ASM remains a topic of ongoing exploration. Despite this complexity, numerous studies have reached conclusions on the intricate process of ASM remodeling, which includes ASM cell proliferation and apoptosis (255, 256), dysregulation Ca2+ homeostasis (257–259), excessive production of ECM components like fibronectin and collagen type I that play a role in stimulating the proliferation of human ASM cells (260–264), and factors associated with immune cells, airway cells, growth factors, cytokine and inflammatory mediators (263, 265–268).

The precise mechanism behind the hypertrophy and hyperplasia of ASM induced by obesity remains insufficiently understood. However, the similarities in the underlying mechanisms between obesity and asthma-induced hypertrophy and hyperplasia of ASM have prompted researchers to explore potential mechanisms responsible for obesity-induced ASM remodeling. One plausible mechanism implicated in obesity-induced ASM remodeling involves the participation of HMGB1. Interestingly, this protein, which has already garnered attention in the context of epithelial remodeling associated with obesity, as discussed previously, may also be associated with obesity-induced ASM remodeling. The administration of HMGB1 substantially increased proliferation, migration, collagen secretion, and expression of α-SMA, with noteworthy findings indicating that the HMGB1/IL-1β complex significantly elevated the expression and secretion of critical regulatory factors, including TGF-β1, MMP-9, and VEGF (263). The mechanism of HMGB1 attenuating ASM proliferation may be related to downstream signaling involving IL-13 and TLR-4. Blocking HMGB1 reduced ASM proliferation and lowered TGF-β expression, indicating that HMGB1 plays a role in ASM remodeling related to TGF-β. Additionally, neutralizing IL-13 reduced HMGB1 levels in cultures of innate lymphoid cells type 2 (ILC2) and ASM stimulated by IL-2/IL33. Blocking HMGB1 or TLR4 activation antagonized the reduced ILC2-induced ASM proliferation, suggesting an association between HMGB1, TLR4, and downstream signaling from IL-13 (269).

The second potential mechanism involves adipose tissue inflammation and the activation of inflammatory responses. Neutrophils, along with neutrophil elastase, a major inflammatory protease released by neutrophils, may contribute to obesity-induced ASM remodeling. Elevated levels of blood neutrophils were observed in individuals with obesity compared to lean individuals, and this increase correlates with BMI (270–272). Furthermore, several studies have reported elevated levels of neutrophil elastase (273–275) and myeloperoxidase (274, 276) in obese animal models and human subjects. Neutrophil elastase stimulates ASM cell proliferation by enhancing cyclin D1 activity through the ERK signaling pathway (277). Neutrophils exert their influence on ASM proliferation by releasing neutrophil-derived exosomes, which promote ASM cell proliferation (278). Another inflammatory factor related to obesity-induced ASM remodeling is the involvement of adaptive immune cells, particularly CD4+ T cells. The number of CD4+ T cells in adipose tissue was higher in obese subjects with metabolically abnormal insulin resistance compared to both obese subjects with metabolically normal insulin sensitivity and lean individuals, suggesting alterations in adipose tissue CD4+ T cells contribute to insulin resistance in individuals with obesity (279). The activation of antigen-specific CD4+ T cells initiates a potent inflammatory response characterized by the infiltration of activated CD4+ T cells into the airway wall. This infiltration leads to a marked increase in ASM mass, primarily driven by enhanced myocyte proliferation and the suppression of baseline apoptosis (266). Airway smooth muscle cell proliferation appears to be mediated primarily by CD4+ T cells, potentially via interactions with the EGFR (265).

Elevated TGF-β1 levels have been consistently observed in patients with obesity, particularly those with comorbidities, as well as in animal models exposed to HFD. These findings align with the observation that TGF-β1 levels exhibit a positive correlation with BMI (231, 280–282). Transforming growth factor-β1 plays a pivotal role in promoting the proliferation of ASM cells by facilitating the accumulation of fibronectin and collagen Type I, key ECM proteins. These ECM proteins then interact with RGD-binding integrins, including the α5β1 integrin (283). Additionally, research has illuminated potential mechanisms involved in TGF-β1-induced ASM remodeling, encompassing pathways such as the phosphorylation of p38 and ERK1/2 (284), the up-regulation of miR-181a and miR-328-3p through the suppression of phosphatase and tensin homolog (PTEN) protein and increased p-Akt/Akt ratio suggesting TGF-β1 induced ASM remodeling involves PTEN and Akt signaling pathway (285, 286), and the modulation of intracellular calcium levels [(Ca2+)i] and/or activation of Rho-associated protein kinase (ROCK) (287).

Interestingly, the role of leukotrienes in obese-induced epithelial airway remodeling, as discussed earlier, also extends to obese-induced ASM remodeling. Leukotrienes and their derivatives play a role in adipogenesis and the inflammation process in adipose tissue (114–116, 288). This suggests that leukotrienes could be a key pathway for inducing ASM remodeling in obese conditions. Leukotriene D4 promotes ASM growth, while the administration of CysLT antagonists inhibits ASM mass growth and reduces ASM hyperplasia, highlighting the crucial role of leukotrienes in ASM remodeling (110, 289). The inhibition of EGFR significantly reduces LTD4-induced ASM hyperplasia, underscoring the involvement of EGFR in ASM remodeling. This finding implies that EGFR and CysLTs play interconnected roles in the complex process of ASM remodeling (289).

The pathological process associated with obesity-induced ASM remodeling may also involve adipocytokines and VEGF. Leptin promotes ASM cell proliferation (290) and significantly inhibits ASM cell apoptosis, partially through the PI3K/Akt signaling pathway (291). In contrast, another study noted that leptin inhibited ASM migration and proliferation (292, 293), yet it did stimulate VEGF release (293). The inconsistent finding suggests that leptin may not directly induce ASM proliferation but could stimulate another factor, such as VEGF, to induce ASM proliferation, as VEGF is known for inducing ASM proliferation and migration (294–296). The remodeling of ASM induced by VEGF may be associated with the activation of the RhoA/ROCK pathway (296). The interaction between leptin and VEGF in obesity-induced ASM remodeling should be considered, as higher levels of serum VEGF were observed in individuals with obesity compared to the control group (297–300).

The pathophysiological role of extracellular vesicles (EVs) in lung disease is attracting growing attention, and the asthma-obesity phenotype may be distinguished by utilizing extracellular EVs for phenotype identification. For instance, EVs miRNA signatures, particularly the miR-17–92 and −106a–363 clusters, could be employed to identify the precise mechanisms underlying the low type-2 asthma phenotype associated with obesity (301). A subpopulation of EVs or exosomes derived from adipose tissue-derived mesenchymal stem/stromal cells (AT-MSCs) have demonstrated beneficial effects in asthma (302–304). On the contrary, the impact of EVs secreted by the respiratory system on adipose tissue remains unclear (305). Studies related to the application of these EVs/exosomes in the context of airway remodeling are still limited; however, they may be related to obesity-induced ASM remodeling. For instance, exosomal miR-301a-3p derived from AT-MSCs suppressed the PDGF-BB-stimulated proliferation and migration of ASM cells by targeting STAT3 (303).

Lipid receptors in the structural components of the airway may be involved in the development of obesity-induced ASM remodeling, with one crucial receptor being free fatty acid receptor 1 (FFAR1), serving as a sensor for medium- and long-chain free fatty acids and expressed on ASM (306, 307). Free fatty acid receptor 1 plays a pivotal role in airway contraction and ASM cell proliferation, as observed in a study demonstrating that FFAR1 promotes ASM cell proliferation through MEK/ERK and PI3K/Akt signaling pathway (308).

Taken together, the process of obesity-induced ASM remodeling is intricate and multifaceted, involving various factors. These include neutrophils, neutrophil elastase, CD4+ T cells, HMGB protein, TGF-β1, leukotrienes, and their derivatives. The interaction between leptin and VEGF may contribute to stimulating ASM remodeling. Additionally, the potential role of extracellular vesicles derived from adipose tissue and lipid receptors such as FFAR1 in airway structure, especially smooth muscle, may also be involved in this mechanism. Further research is needed, and its findings could have significant implications for the management and treatment of airway remodeling in individuals with obesity and asthma.

### 4.5 Obesity-associated bronchial vascular airway remodeling in asthma

Several key factors are implicated in the initiation and progression of bronchial vascular remodeling, including the pro-inflammatory cytokines (309–311), angiopoietin (312, 313), and growth factors specifically VEGF (314–316). Vascular remodeling is more pronounced in the advanced stages of GINA treatment and is associated with airflow obstruction (314).

Vascular endothelial growth factor emerges as a potentially pivotal factor in the development of obesity-induced bronchial vascular remodeling in individuals with asthma, acting as a key regulator of angiogenesis and governing various endothelial functions, including proliferation, migration, vascular permeability, and secretion (317). Vascular endothelial growth factor is generated not solely by endothelial cells but also by diverse cell types within the respiratory system, including epithelial cells (318, 319), ASM cells (320), CD4+ T cells (321), alveolar type 2 cells (322), and alveolar macrophages (323). Among the VEGF subfamily members, VEGF-A is particularly crucial, and its receptors, VEGFR-1 (Flt-1) and VEGFR-2 (KDR/Flk-1) play vital roles in processes like angiogenesis, vascular permeability activity, and cell migration (317, 324). Elevated serum VEGF levels were observed in obese patients and this elevation has associations with visceral fat index and BMI (297, 300, 325). Meanwhile, overweight individuals with asthma exhibited higher levels of VEGF-A compared to their non-overweight counterparts (326). Interestingly, the animal study observed that obesity itself, as well as the asthma and obese-asthma groups, enhance mRNA VEGF expression and elevate VEGF in BAL cytokines compared to the control group (327). Moreover, elevated levels of leptin, a prevalent occurrence in individuals with obesity, have been shown to stimulate the secretion of vascular endothelial growth factor (VEGF) by airway ASM cells (293), suggesting that leptin may indirectly contribute to the obesity-induced bronchial vascular remodeling processes in individuals with asthma and obesity.

Angiopoietin-2 may emerge as a significant contributor to the development of obesity-induced vascular remodeling, as angiopoietins play crucial roles in regulating vascular remodeling in endothelial cells. Elevated angiopoietin-2 was associated with capillary enlargement rather than angiogenesis (312), suggesting that angiopoietin-2 primarily affects endothelial cell proliferation. However, in a separate study, angiopoietin-2 also plays a role in angiogenesis by mediating the angiogenic process in pulmonary endothelial cells (328). Significantly elevated levels of angiopoietin-2 were observed in individuals with asthma compared to healthy subjects, and these levels correlated with the severity of asthma (329). Meanwhile, increased angiopoietin-2 levels were also observed in individuals with overweight and obesity compared to lean control subjects (300, 330), further indicating the potential involvement of angiopoietin-2 in obesity-induced vascular remodeling. On the other hand, angiopoietin-2 may contribute to the pathological process in adipose tissue. Both subcutaneous and epididymal white adipose tissues experience a notable upregulation in angiopoietin-2 expression, leading to increased vascular density. Conversely, neutralizing angiopoietin-2 results in a reduction in blood vessel formation within adipose tissue, triggering pro-inflammatory and pro-fibrotic changes (331). The dual role of angiopoietin-2 in the pathological processes in the airway and adipose tissue has the potential to be considered an important factor in obesity-induced vascular remodeling.

An additional potential mechanism contributing to obesity-induced vascular remodeling could be the deficiency of adiponectin. Adiponectin-deficient mice have shown increased thickening of vessel walls, resulting in near obliteration of the lumen, suggesting a role for adiponectin in vascular remodeling (332). The role of adiponectin in vascular alterations may be related to chronic hypoxia conditions. Hypoxia can trigger pulmonary vascular remodeling (333, 334). Meanwhile, the overexpression of adiponectin has demonstrated a reduction in hypoxia-induced airway wall thickening (335), suggesting that adiponectin may play a role in modulating pulmonary vascular changes related to chronic hypoxia involvement.

Taken together, obesity appears to have the potential to initiate or exacerbate the development of bronchial vascular airway remodeling. While the exact mechanism behind this phenomenon remains inadequately understood and needs to be fully elucidated, several factors have emerged as potential contributors, including the role of VEGF, the involvement of angiopoietin-2, adiponectin deficiency, along hypoxic conditions. The complex interplay of these factors underscores the intricate nature of obesity-induced bronchial vascular remodeling and highlights the need for further research to unravel its underlying mechanisms.

## Conclusion

In conclusion, it is well established that obesity is associated with poor clinical outcomes in individuals with asthma, potentially serving as one of the factors that induce airway remodeling in asthma. Clinical studies investigating the development of airway remodeling in obese individuals are still limited. However, several factors may contribute to the development of airway remodeling in obese individuals. Obesity-induced epithelial remodeling may involve various factors, including HMGB1, the role of leptin, CysLTs, ORMDL3, matrikines like PGP, and cytokines such as IL-4 and IFN-γ, which may contribute to epithelial alterations. The role of chronic inflammatory processes in the development of obesity-induced airway remodeling is still a subject of debate. Several factors, such as oxidative stress, dysregulation of adipocytokines, the inflammasome, anti-inflammatory modulators like secretoglobin and IL-37, and the presence of other comorbidities inducing systemic inflammation may contribute to the development of inflammatory conditions in individuals with asthma and obesity. Obesity may also play a role in the development of airway fibrosis, contributed to by factors such as TGF-β1, MMP-9, and FoxO1. The development of obesity-induced ASM remodeling is an intricate process influenced by factors like neutrophils, neutrophil elastase, CD4+ T cells, HMGB1 protein, TGF-β1, leukotrienes, and their derivatives. Additionally, the interaction between adipocytokines and VEGF, extracellular vesicles, and lipid receptors on smooth muscle can contribute to this complex process. Obesity may also contribute to airway vascular remodeling, influenced by factors such as VEGF, the role of leptin, deficiency in adiponectin, and angiopoietin. Although several factors may be potentially related to obesity-induced airway remodeling, clinical and laboratory studies are needed to elucidate the exact mechanisms of how obesity impacts airway remodeling in asthma. This can be beneficial in preventing the development of airway remodeling and implementing a personalized approach to management in patients with asthma and obesity.
